# Gendered racial microaggressions, internalized racism, and suicidal ideation among emerging adult Asian American women

**DOI:** 10.1177/00207640221089536

**Published:** 2022-04-12

**Authors:** Brian TaeHyuk Keum, Michele J. Wong, Rangeena Salim-Eissa

**Affiliations:** Department of Social Welfare, University of California, Los Angeles, USA

**Keywords:** Asian American women, gendered racial microaggressions, internalized racism, suicidal ideation

## Abstract

**Background:**

Despite suicide being the leading cause of death among emerging adult Asian American women (AAW), little is known about the risk factors.

**Aim:**

We tested whether gendered racial microaggressions stress (GRMS) would be associated with AAW’s suicidal ideation, and whether internalized racism (self-negativity, IRSN; weakness stereotypes, IRWS; and appearance bias, IRAB) would exacerbate this link based on self-devaluating implications of internalized racism.

**Method:**

Using a sample of 309 AAW (*M*_age_ = 20.00, *SD* = 6.26), we conducted a moderated logistic regression with GRMS predicting suicidal ideation (endorsement or no endorsement) and the three internalized racism factors (IRSN, IRWS, and IRAB) as moderators.

**Results:**

GRMS significantly predicted suicidal ideation with a threefold increase in the odds of suicidal ideation. Only IRSN significantly exacerbated this link at low to mean levels.

**Conclusion:**

Gendered racial microaggressions is likely a risk factor for suicidal ideation among AAW, particularly for those who internalize negative images of themselves as Asian individuals.

The risk for suicide has been increasing among Asian American women (AAW). A 2009 study found that U.S.-born AAW had a higher lifetime rate (15.9% vs. 13.5%) of suicidal thoughts compared to the general population (Duldulao et al., 2009). Alarmingly, suicide is the leading cause of death among AAW 15 to 24 years old (Centers for Disease Control and Prevention [CDC], 2019). In response, researchers have begun to explore the complexities of suicidality among AAW, including stress from racial stereotypes such as the model minority myth that can push them to consider suicide (Noh, 2018).

Extant literature has linked perceived racial discrimination to suicidal ideation among diverse groups of Asian Americans (Cheng et al., 2010; Kuroki, 2018; Leong et al., 2007). However, no studies have examined the potential suicide risk associated with the specific lived experiences and internalization of oppression that AAW face. Recognizing the unique interlocking forms of oppression based on their ‘Asianness’ and ‘femaleness’, researchers have begun to explore the experiences and negative health impacts of gendered racism among AAW (Keum et al., 2018a; Mukkamala & Suyemoto, 2018). Thus, the purpose of the current study was to (a) examine the link between gendered racial microaggressions and suicidal ideation among AAW and (b) test internalized racism as a potential moderator of this link.

## Gendered racial microaggressions and suicidal ideation

Gendered racial microaggressions (GRM) is defined as everyday expressions and exchanges, regardless of intention, that denigrates individuals based on their intersecting gender and racial identities (Crenshaw, 1989; Lewis & Neville, 2015). Keum et al. (2018a) operationalized GRM for AAW as comprising four domains: (a) Ascribed Submissiveness (i.e. treating AAW as if they will always comply and be submissive), (b) Assumption of Universal Appearance (i.e. restrictive and stereotypical appearance expectations), (c) Asian Fetishism (e.g. sexual objectification, ‘yellow fever’), and (d) Media Invalidation (e.g. lack of AAW representation or stereotypical portrayals). GRM (total score) predicted variance in depressive symptoms among AAW above and beyond general racial microaggressions and sexism variables, suggesting a unique risk for AAW’s mental health issues (Keum et al., 2018a).

GRM may be a salient risk factor for suicidal ideation among AAW based on the Interpersonal-Psychological Theory of Suicide (IPTS; Joiner, 2007). According to IPTS, the desire for suicide (i.e. suicidal ideation) is driven by a sense of hopelessness stemming from thwarted belongingness (i.e. unmet need for social connectedness) and perceived burdensomeness (i.e. perception that one is considered a strain on their family, friends, and/or society; Joiner, 2007). In a narrative analysis among AAW, Noh (2018) found that racist and/or sexist work culture, along with tremendous pressure from model minority expectations to perform well, made women feel devalued, objectified, and were the conditions that ultimately drove them to consider suicide. AAW can feel alienated from others due to these oppressive dynamics, seeing themselves to be a burden to society unless they behaved according to the stereotypes of AAW as submissive, fetishized, and domesticated women (Ma et al., 2016; Noh, 2018; Wong et al., 2011). This marginalizing and self-deprecating dynamic may also be exacerbated within the family, where AAW’s gendered responsibilities to perform both the physical and emotional labor as caretakers often place the needs of others before themselves (Hahm et al., 2014; Noh, 2018). For AAW who internalize the GRM experiences that denigrate their sense of self (e.g. internalized racism), these negative interpersonal states likely contribute to increased feelings of self-hate or self-negativity that ultimately increase the risk for suicide ideation (Turnell et al., 2019).

## Internalized racism as a potential moderator between GRMS and suicidal ideation

Internalized racism has been associated with negative psychosocial outcomes among Asian Americans (David et al., 2019; Gale et al., 2020; Garcia et al., 2019). Defined as the adoption or acceptance of oppressive actions, beliefs, and values of dominant White culture about racial minorities, internalized racism can manifest through self-hatred, negative stereotypes, discrimination, racist doctrines, and White supremacy beliefs (Choi et al., 2017). For example, a qualitative study on East and Southeast Asians found that U.S.-born individuals would use labels like ‘fresh off the boat’, to describe their foreign-born co-ethnic peers to resist the racially stigmatized status as perpetual foreigners. This is one prime example of how internalized racism and idealization of Whiteness lead to in-group discrimination and negative stereotyping to reproduce racial hierarchies (Pyke & Dang, 2003).

Internalized racism has also been posited as a survival mechanism for Asian Americans living in a ‘White racial frame’ where all aspects of life are permeated by racist imagery and stereotypes, and achieving a certain level of Whiteness may even be considered healthy for some (Chou & Feagin, 2015; Millan & Alvarez, 2014). Unfortunately, reinforcing one’s own oppression by internalizing dominant narratives that perpetuate Asian American inferiority can prove to be more detrimental than beneficial (Choi et al., 2017; David et al., 2019). Garcia et al. (2019) found that higher levels of internalized racial inferiority intensified the link between racism and psychological distress among adult Asian Americans. Internalized misogyny, another form of internalized oppression, has also been found to amplify the link between women’s sexist experiences and their psychological distress (Szymanski et al., 2009). These results suggest internalized racism as a potential moderator of GRM’s role on suicidal ideation among AAW.

Choi et al. (2017) operationalized three dimensions of general and gendered forms of internalized racism for Asian Americans: (a) self-negativity, (b) weakness stereotype, and (c) appearance bias. Self-negativity refers to the wholesale devaluation of one’s own Asian American identity, reflected in the desire to be part of the White dominant group, expression of negative attitudes about being an Asian, and low collective self-esteem in being part of the Asian community (Choi et al., 2017). Weakness stereotypes refer to the internalization of Asian Americans as inherently weak, less assertive, and incompetent due to some immutable racial flaw (Choi et al., 2017). Compared to Asian women, weakness stereotypes may carry a heavier burden among Asian men as they are often portrayed as effeminate, emasculated, and fall short of the White hegemonic masculinity ideals (Liu & Wong, 2018). By comparison, the internalization of weakness stereotypes may align with the racialized and gendered assumptions of Asian women as diminutive, meek, hyperfeminine, and exoticized (Keum et al., 2018a). Especially for AAW who feel the pressure to subscribe to hyperfeminine and traditional femininity norms (Mukkamala & Suyemoto, 2018), internalizing these stereotypes may have complex implications on their mental health and identity development. Similarly, appearance bias which describes self-devaluation of Asian phenotypic features (Choi et al., 2017), may also have mixed implications on AAW’s well-being given the deep-seated preference for White attractiveness ideals in the Asian communities (Brady et al., 2017; Wong et al., 2017). Wong et al. (2017) found that AAW receive messages reinforcing White attractiveness ideals (e.g. paler skin) from their peers (including other AAW), men, parents, and communities, and are often rewarded for working toward these ideals (Brady et al., 2017). These AAW reported a dilemma in which they find themselves needing to adhere to White beauty standards despite their awareness of damaging and self-devaluing body image perceptions (Wong et al., 2017). Overall, the literature portrays clarity on self-negativity potentially exacerbating the risk among AAW while the role of appearance bias and weakness stereotypes may be more complex.

## The present study

We tested the link between GRM and suicidal ideation among emerging adult AAW and the three factors of internalized racism (self-negativity, weakness stereotype, and appearance bias) as moderators. Emerging adult AAW likely exhibit certain levels of internalized racism that may affect the link between GRM and thoughts of suicide. As reviewed, it is possible that for AAW who buy into the weakness and appearance-related stereotypes (e.g. sexualized and submissive images), they may engage in this process with resigned acceptance or as a willing choice to survive and to align with the stereotypical expectations and White cultural norms in the United States (Cheng & Kim, 2018). While this process still reflects a stressful adjustment and self-erasure, it may provide AAW with an illusionary sense of feeling included and adjacent to the White mainstream society (e.g. honorary Whites; Tinkler et al., 2019). On the other hand, AAW who particularly internalize the GRM experiences as self-negative, and inferior self-concepts (e.g. ‘Compared to Whites, I am less than, less worthy’), their feelings of alienation and undesirability may be exacerbated and place them at higher risk for lacking the desire to live. Accordingly, we tested the below hypotheses:

**Hypothesis 1.** Higher GRM stress would be significantly related to higher suicidal ideation among AAW.
**Hypothesis 2.** Internalized racism factors would moderate the link between high GRM stress and high suicidal ideation such that:
  (A) Higher internalized self-negativity would strengthen this relationship.
  (B) Higher internalized weakness stereotype and appearance bias would weaken this relationship or have a non-significant interaction effect.

Given that generational status may impact the awareness of discrimination among Asian individuals (Keum et al., 2018c) and age likely influence some of the body-related perceptions represented the GRM (Keum, et al., 2018a), we controlled for generational status and age. Additional covariates were included based on statistical relevance.

## Method

### Participants

Data were collected from a non-probability sample of 309 AAW (*M*_age_ = 20.00, *SD* = 6.26). The sample was diverse with regards to ethnicity: Chinese (28.5%, *n* = 88), Multiracial/Multiethnic (17.2%, *n* = 53), Korean (16.2%, *n* = 50), Indian (10%, *n* = 31), Vietnamese (6.8%, *n* = 21), Taiwanese (6.8%, *n* = 21), Filipino (5.8%, *n* = 18), and other (8.6%, *n* = 27). Most participants identified as women (99.7%, *n* = 308), and one participant (0.3%) identified as genderfluid. About 82.5% (*n* = 255) identified as heterosexual, 6.5% (*n* = 20) bisexual, 2.3% (*n* = 7) uncertain, 2.3% (*n* = 7) as queer, 1.9% (*n* = 6) asexual, 1.6% (*n* = 5) questioning, 0.3% (*n* = 1) as lesbian, 0.3% (*n* = 1) as gay, and 2.3% (*n* = 7) as other. About 68.6% (*n* = 212) identified as second generation (native born with at least one immigrant parent), 10.7% as first generation (born outside the U.S.), 4.5% (*n* = 14) as 1.5 generation (immigrated to U.S. ages 6–12), 4.5% (*n* = 14) as third generation and beyond (native born, at least both parents born in U.S.), 3.9% (*n* = 12) as 1.75 generation (immigrated to U.S. ages 0–5), 2.9% (*n* = 9) as 1.25 generation (immigrated to U.S. ages 13–17), 2.6% (*n* = 8) as adoptee, and 2.3% (*n* = 7) as other.

### Measures

#### Internalized racism

The Internalized Racism in Asian American Scale (IRAAS; Choi et al., 2017) is a 14-item, three-factor scale that aims to measure the level of internalized negative messages regarding Asian American racial identity. The three factors are: (a) Self-Negativity; global devaluation and negative attitudes toward one’s own Asian American identity, (b) Weakness Stereotypes; internalized beliefs of deficit or weakness inherent to being Asian American, and (c) Appearance Bias; devaluation of Asian appearances. Items are rated on a 6-point Likert scale (1 = *strongly disagree* to 6 = *strongly agree*). Higher scores indicate greater internalized racism. A sample item is ‘My life would be better if I wasn’t Asian’. Convergent validity and predictive validity were demonstrated with Asian Americans (Choi et al., 2017). The internal consistency estimates were 0.80, 0.84, and 0.76 for IRSN, IRWS, and IRAB, respectively.

#### Gendered racial microaggressions stress

The Gendered Racial Microaggressions Scale for Asian American Women (GRMSAAW; Keum et al., 2018a) is a 22-item, bifactor (four specific factors) scale that assesses the behavioral, verbal, and environmental manifestations of GRM experienced by AAW in the United States. The four subscales are: (a) Ascribed Submissiveness; microaggressions rooted in submissive stereotypes and assumptions of AAW, (b) Assumption of Universal Appearance; stereotypes and assumptions that minimize and confine all AAW’s body image and appearance attributes to certain ‘Asianized’ standards, (c) Asian Fetishism; sexual objectification and fetish (e.g. yellow fever), and (d) Media Invalidation; underrepresentation and negative stereotypical portrayals in the media. The general factor (total score) represented a unique shared variance across all items. We used the stress appraisal total scale (GRMS) with items rated on a 6-point Likert scale (0 = *not at all stressful* to 5 = *extremely stressful*). Higher scores indicate greater GRMS. Sample items include ‘Others express sexual interest in me because of my Asian appearance’, and ‘I rarely see AAW in the media’. Keum et al. (2018a) reported good internal consistency with Cronbach’s alphas ranging from .86 to .94 for stress appraisal. Construct validity was supported by associations with racial microaggressions, sexism, depression, and internalized racism scores (Keum et al., 2018a). The total score internal consistency estimate for the current study was 0.90.

#### Suicidal ideation

Patient Health Questionnaire-9 (PHQ-9; Kroenke & Spitzer, 2002) is a 9-item depression scale that establishes provisional depressive disorder diagnoses and depressive symptom severity based on responders’ symptoms reports in the past 2 weeks. We used item nine (‘I have thoughts of ending my life’) to assess suicidal ideation. We dichotomized participants’ scores: 0 indicating no suicidal ideation (i.e. those who rated ‘not at all’) and 1 indicating the presence of suicidal ideation (i.e. those who rated ‘several days’, ‘more than half the days’, and ‘nearly every day’). Scores on the PHQ-9 were correlated with the Symptom Checklist-20 (Kroenke & Spitzer, 2002). Validity and measurement invariance of PHQ-9 with Asian American college students has been supported (Keum et al., 2018b).

### Procedure

The study was approved by the Institutional Review Board. Participants were invited to take an online survey hosted by Qualtrics which was advertised through online communication strategies including e-mail (e.g. listservs), discussion forums, and online social networks catering to Asian women residing in the U.S. (e.g. Facebook pages and Google groups). The online survey consisted of study variable measures and demographic items. The inclusion criteria for the study were: (1) 18 years old or older, (2) self-identify as an Asian/Asian American woman, and (3) live in the United States. Informed consent was provided and obtained from all participants. The survey took 15 to 20 minutes to complete and included two attention check items (e.g. ‘Please choose always’).

### Data analysis

The data were analyzed using the PROCESS macro in SPSS 24 (Hayes, 2018). Bivariate correlations and descriptive statistics of the study variables were first assessed. We selected and ran Model 1 in PROCESS macro version 3.5 for SPSS (Hayes, 2018) to conduct a moderated logistic regression between GRMS (IV) and binary outcome of suicidal ideation (DV; (0 = no ideation, 1 = thoughts of suicide several days or more) with bias-corrected bootstrapping (10,000 resamples). The three IRAAS factors were entered as moderators. Age, generational status, and other statistically relevant demographic factors were entered as covariates. With a binary DV, PROCESS generates results in log-odds metrics. Log-odds were converted into odds ratios for ease of interpretation. PROCESS uses an ordinary least squares regression-based path analytic framework for estimating the interactions in moderation models along with simple slopes for probing the interactions. The significance of the simple slopes was assessed if the interaction was significant. We used Ferguson’s (2016) criteria to assess effect sizes of the *r* and path coefficients, in which .20 is the recommended minimum effect size representing a practically significant effect, .50 represents a moderate effect and .80 represents a large effect.

## Results

### Data inspection and preliminary analysis

Little’s missing completely at random (MCAR) test was not significant, suggesting that data were missing completely at random, χ^2^ (140) = 167.94, *p* = .054. Of the 506 cases, 197 were missing more than 20% of the data and were removed. In the remaining sample, five cases were missing less than 10% of the data. We used the expectation maximization estimates for multiple imputations of the missing values. We computed the mean or total scores for the corresponding scales in our analysis. The final sample size was 309.

Descriptive statistics and bivariate correlations of study variables are listed in Table 1. About 20% (*n* = 62) of the participants reported suicidal ideation, in line with the prevalence trends among Asian Americans (Cheng et al., 2010). Bivariate correlations suggested that suicidal ideation was significantly correlated with the independent variables at small effect. The VIF values for all the independent variables ranged from 1.15 to 1.88, all of which were within the 1 to 10 range suggesting evidence of no multicollinearity (George & Mallery, 2010). Thus, the assumption of little or no multicollinearity for logistic regression was satisfied. In addition to controlling for age and generational status as theory-driven covariates, SES was also controlled as it was significantly correlated with GRMS and IRSN.

**Table 1.** Descriptive statistics and bivariate correlations.

	1	2	3	4	5	6	7	*M*	*SD*	Range
1. GRMS	—							3.77	0.97	0–5
2. IRSN	.24**	—						2.57	1.35	1–6
3. IRWS	−.08	−.08	—					2.64	1.18	1–6
4. IRAB	−.12	−.47**	.64**	—				2.07	1.13	1–6
5. Suicidal ideation	.12	.27**	.12*	.21*	—			0.20	0.40	0–1
6. Age	.12*	−.05	−.08	−.12*	−.13*	—		20.00	6.26	18–67
7. Generational status	.07	−.09	.004	−.04	−.001	−.07	—	4.50	1.54	
8. SES	.12*	.13*	−.001	.04	.02	.05	−.13*	3.05	1.02	

*Note*. GRMS = gendered racial microaggressions stress; IRSN = internalized racism self-negativity; IRAB = internalized racism appearance bias; IRWS = internalized racism weakness stereotype; SES = socioeconomic status.

* *p* < .05. **p < .01.

### Moderated logistic regression

The results of the moderated logistic regression model are listed in Table 2. After controlling for age, generational status, and SES, the main effects of GRMS and IRSN were significant. Higher GRMS was significantly associated with suicidal ideation, log odds = 1.08, Odds Ratio = 2.95, 95% CI [1.27, 6.82], *SE* = 0.43, *p* = .012). Thus, a one unit increase in GRMS suggests 2.95 times higher odds of AAW endorsing suicidal ideation. Higher IRSN was also significantly associated with suicidal ideation, log odds = 1.71, Odds Ratio = 5.55, 95% CI [1.75, 17.64], *SE* = 0.59, *p* = .004); a one unit increase in IRSN suggests 5.55 times higher odds of AAW endorsing suicidal ideation.

**Table 2.** Moderated logistic regression model predicting suicidal ideation.

	Log-odds	*SE*	*p*-Value	CI	OR
Constant	−5.26	2.11	.013	[−9.40, 1.13]	0.01
GRMS	1.08	0.43	.012	[0.24, 1.92]	2.95
IRSN	1.71	0.59	.004	[0.56, 2.87]	5.55
IRAB	−0.98	0.71	.170	[−2.37, 0.41]	0.38
IRWS	−0.001	0.69	.999	[−1.36, 1.36]	0.99
GRMS × IRSN	−0.37	0.17	.022	[−0.69, −0.05]	0.69
GRMS × IRAB	0.30	0.17	.071	[−0.03, 0.63]	1.35
GRMS × IRWS	−0.05	0.16	.748	[−0.37, 0.27]	0.95
Age	−0.07	0.04	.058	[−0.14, 0.002]	0.93
Generational status	0.02	0.11	.860	[−0.19, 0.23]	1.02
SES	−0.11	0.16	.510	[−0.42, 0.21]	0.90

*Note.* GRMS = gendered racial microaggressions stress; IRSN = internalized racism self-negativity; IRAB = internalized racism appearance bias; IRWS = internalized racism weakness stereotype; SES = socioeconomic status; CI = confidence intervals, OR = odds ratios.

**Figure 1.**
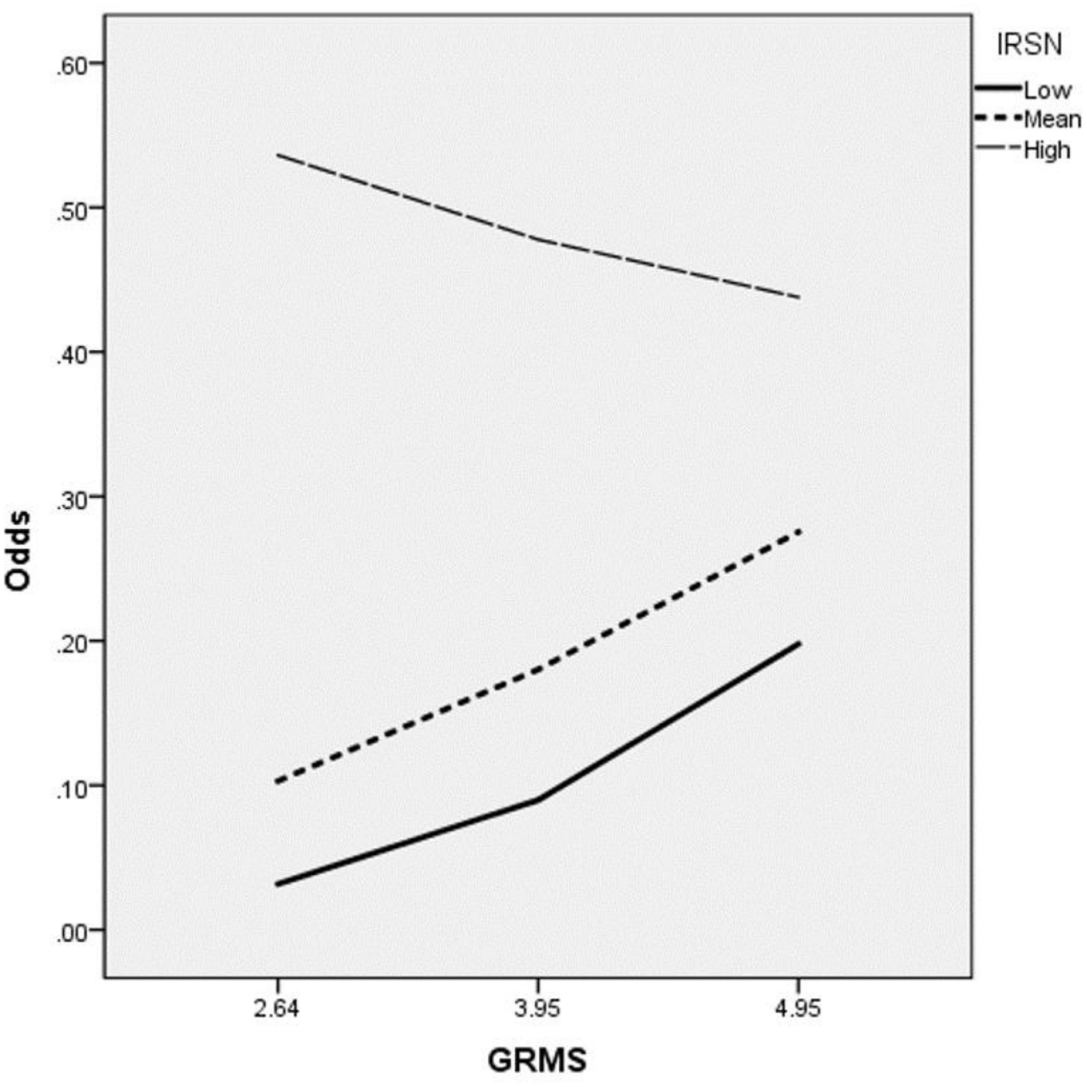
Internalized racism self-negativity as a moderator between gendered racial microaggression stress and suicidal ideation. *Note*. IRSN = internalized racism self-negativity; GRMS = gendered racial microaggression stress; *Y*-axis = odds ratios of suicidal ideation.

The GRMS × IRSN interaction term was significant (*b* = −0.29, *SE* = 0.13, *p* = .028). Simple slopes at mean and 1 *SD* above/below the mean levels of IRSN were assessed to examine the significance of the interaction. Figure 1 presents the slopes with suicidal ideation probabilities on the *Y*-axis and GRMS on the *X*-axis. Slopes were positive and significant at low (−1 *SD* below the mean; *b* = 0.79, *SE* = 0.31, *p* = .011, Odds Ratio = 2.20, 95% CI [1.20, 4.04]) and mean IRSN levels (*b* = 0.42, *SE* = 0.18, *p* = .022, Odds Ratio = 1.53, 95% CI [1.07, 2.19]), suggesting that the probability of endorsing suicidal ideation increases significantly with greater GRMS. At 1 *SD* above the mean (high IRSN), slopes were not significant, (*b* = −0.09, *SE* = 0.20, *p* = .664, Odds Ratio = 0.92, 95% CI [0.62, 1.36]), suggesting that the probability of endorsing suicidal ideation did not change significantly at high levels of IRSN.

As seen in Table 2, neither IRAB (*b* = −0.98, *SE* = 0.71, *p* = .166) nor IRWS (*b* = −0.001, *SE* = 0.69, *p* = .999) was significantly related to suicidal ideation. As hypothesized, neither IRAB (*b* = 0.30, *SE* = 0.17, *p* = .071) nor IRWS (*b* = −0.05, *SE* = 0.16, *p* = .748) was a significant moderator.

## Discussion

This is the first study to examine the risk of suicidal ideation associated with GRM that denigrates and invalidates the gendered and racialized identities of AAW (Keum et al., 2018a). In line with our hypothesis, GRM stress significantly predicted suicidal ideation among AAW; in fact, a one unit increase in GRM stress indicated a threefold increase in the odds of endorsing suicidal ideation. Our examination of internalized racism provided some complicated gendered intricacies, as only the internalization of self-negativity (and not the appearance bias and weakness stereotype factors) exacerbated greater suicidal ideation.

According to the IPTS, the desire for suicide arises from feelings of thwarted belongingness and perceived burdensomeness that is further motivated by a sense of hopelessness and a capability for suicide (Chu et al., 2017; Joiner, 2007). Although these two variables were not tested in our study, as we elaborated, GRM is likely an everyday social burden that gives rise to these aspects. AAW who frequently experience GRM are likely to deal with feelings of loneliness, alienation, undesirability, self-hate, and self-devaluation that increase the risk of suicidal ideation (Noh, 2018; Wong et al., 2011). These indicators of suicidal ideation may often go overlooked in clinical settings, schools, and community spaces, especially as AAW continue to experience the repercussions of the model minority myth that assumes unmitigated success and belies the mental health issues they face (Noh, 2018). With suicide rates continuing to increase among AAW (CDC, 2019; Noh, 2018), it is crucial that research continues to examine the interplay between GRM and suicidal ideation in this under-researched and overlooked population.

Of the three internalized racism factors, only IRSN was a significant moderator that exacerbated the link between GRM stress and suicidal ideation. This is consistent with Garcia et al. (2019) which found that the relationship between racial/ethnic discrimination and psychological distress was strengthened at all levels of internalized racism. Interestingly, at high levels of IRSN, there were no significant changes in the probability of having suicidal ideation among AAW (no significant moderation). However, it appeared that those with high levels of IRSN may generally be endorsing a higher probability of suicidal ideation than those with lower IRSN. Upon closer examination, about 40% of the AAW at 1 *SD* above the mean or greater levels of IRSN endorsed suicidal ideation while about 16% endorsed SI among AAW reporting IRSN lower than this range. AAW that report consistently high levels of self-negativity may already harbor considerable negative attitudes about themselves and even suicidal thoughts regardless of the extent to which they experience gendered racism.

Along with the non-significant moderation results on IRWS and IRAB, the findings highlight the complexity of the interplay between GRM and internalized racism among AAW when considering their impact on suicidal ideation. As noted in previous internalized racism studies with people of color (e.g. Sosoo et al., 2020), suicidal ideation in the context of GRM may vary depending on AAW’s level of acceptance and internalization of the dominant White culture’s stereotypes and beliefs toward AAW. AAW who experience greater self-negativity may feel as though they are in a double bind, as they work to conform to White expectations of how they should appear (e.g. hypersexualized images) and present themselves (e.g. weak and submissive) in a bid to fit in and reduce feelings of marginalization from the mainstream White culture (Mukkamala & Suyemoto, 2018; Yamamoto, 2000). This experience of being pulled into multiple directions represents a ‘fractured reality’ of navigating model minority myth expectations for success but living with a ‘fractured identity’ that reflects the inability to integrate the expectations, norms, and obligations of opposing cultural worlds (e.g. school vs. home), and eventually induce feelings of low self-worth (Hahm et al., 2014; Noh, 2018).

### Limitations and future directions

Despite the strengths of our study, there are several noteworthy limitations. First, we used cross-sectional data that precludes us from considering any causal implications of the results. Although much of the literature points to identity-based discriminatory events as a significant precursor to developing suicidal ideation (e.g. Kuroki, 2018), the temporal sequence of our findings should be explored in a future study with longitudinal data. Second, most of our sample was comprised of heterosexual, second generation AAW with East Asian roots. While some of the stereotypes in the United States about AAW may be shared across different Asian ethnicities, our study is limited in generalizability beyond the major identities represented in the sample. For instance, some of the body-related aspects of the GRM and internalized racism may be more representative of East Asian AAW and not as applicable to AAW of South and Southeast Asian backgrounds (Poolokasingham et al., 2014). Furthermore, AAW who spent less time acculturating in the United States may have different perceptions and identifications with racism (Keum et al., 2018b), and sexual minority AAW may experience additional intersecting layers of oppression (Balsam et al., 2011). Thus, future studies should incorporate a more representative sample and consider additional intersecting identities and contexts that is culturally-sensitive to disparities among Asian ethnicities. Third, suicidal ideation was measured using a single item. Although single item measures do provide information on sensitive topics such as suicidality and have been used in other nationally representative studies (Jackman et al., 2019), they are limited in their validity. Future studies should replicate and extend our findings using rigorous measures of suicidality. Finally, the IRAAS was not developed within an intersectionality framework. In addition to employing an intersectional measure of internalized gendered racism, future studies should explore factors that are more proximal to the trigger of suicidal thoughts such as GRM-related feelings of thwarted belongingness, burdensomeness, and self-negativity.

Ultimately, more research is needed to understand how dimensions of internalized racism, particularly appearance bias and weakness stereotypes, may drive a sense of fragmentation and serve a complex survival function among AAW with implications on their mental health and suicide risk. While some AAW may strive for White standards of beauty and attractiveness, others may rely on the ‘othered’ forms of exoticized beauty (Cheng, 2000, p. 46), which produces its own form of capital through the performance of Asian femininity (Cottom, 2019). For instance, Cheng (2019) provides ornamentalism as an alternative conceptual framework that accounts for the complicated history and process of AAW racialization in the United States and suggests that AAW that have come to know themselves through objectifying and flattening stereotypes can still find ways to assert their own sense of agency and autonomy. Future research should integrate critical race theories to conceptualize an alternative understanding of the mechanisms that AAW use to cope with damaging stereotypes that could increase the risk for suicidal ideation.
